# Marionette Line Correction Using Threads

**DOI:** 10.1055/a-2923-5767

**Published:** 2026-10-05

**Authors:** Gi-Woong Hong, Jovian Wan, Song-Eun Yoon, Kyu-Ho Yi

**Affiliations:** 1 Department of Plastic SurgerySamskin Plastic Surgery ClinicSeoulKorea (the Republic of); 2 Department of AestheticMedical Research IncWonjuKorea (the Republic of); 3 Division of AestheticBrandnew Aesthetic Surgery ClinicSeoulKorea (the Republic of); 4 Division in Anatomy and Developmental BiologyDepartment of Oral BiologyHuman Identification Research Institute, BK21 FOUR Project, Yonsei University College of DentistrySeoulKorea (the Republic of); 5 Department of Aesthetic MedicineYou and I ClinicSeoulRepublic of Korea

**Keywords:** cosmetic, facelift, thread, lifting, marionette line, jowl, Asian anatomy, polydioxanone

## Abstract

Marionette lines and jowl ptosis present unique challenges in Asian populations due to distinct anatomical variations. The modiolus in Asians is positioned approximately 11 mm lateral and 9 mm inferior to the oral commissure, compared to at or above the commissure in Caucasian and African populations, predisposing to downturned oral commissures and age-related ptosis. This innovation describes a dual-technique approach combining multidirectional barbed threads for deep structural lifting with volumizing monofilament threads for superficial rhytide correction. Barbed threads are anchored at Loré's fascia in the sub-superficial musculoaponeurotic system plane, engaging ptotic jowl tissues with vectors perpendicular to the marionette line. This combined strategy addresses both deep ptosis and superficial lines while preserving natural facial dynamics and avoiding critical perioral neurovascular structures. The technique offers a minimally invasive solution tailored to Asian facial anatomy.

## Introduction

Facial aging in the perioral region exhibits significant ethnic variations requiring tailored approaches. 1 2 In Asian populations, the inferolateral positioning of the modiolus creates unique challenges for addressing marionette lines and jowl ptosis. 3 4 The modiolus—a dense muscular confluence of perioral muscles—sits approximately 11 mm lateral and 9 mm inferior to the oral commissure in Asians, contrasting with its superior or level position in Caucasian and African populations. 3 This anatomical difference results in a resting downturned commissural orientation and increased susceptibility to age-related ptosis.

The laminar organization of modiolar constituents, particularly the superficial depressor anguli oris (DAO) and intermediate orbicularis oris fibers, creates distinct tissue planes. 4 5 When accentuated, these manifest as the commissural line—a vertical oblique depression particularly prevalent in Asians. 6 Volumetric deficiency in the lateral lower lip fat compartment further exacerbates the disparity between jowl fat and labial tissues, 4 5 contributing substantially to oral commissure ptosis and commissural line prominence.

Progressive tissue thickness disparities between cheek and lower lip regions create the melolabial fold (cheek–chin crease), which extends to the mandibular border with aging. 7 Concomitant prejowl sulcus formation creates the composite marionette line complex. Contributing factors include bone resorption, soft tissue descent, deep fat atrophy, DAO compression, mandibular ligament tethering, and jowl fat ptosis—necessitating multimodal therapeutic strategies. 5 7

Thread-lifting procedures using polydioxanone (PDO) threads offer minimally invasive solutions for these concerns. 8 This paper presents an innovative dual-technique approach specifically designed for Asian facial anatomy, combining barbed threads for structural repositioning with monofilament threads for superficial enhancement. 9 10

## Idea

### Patient Selection and Preparation

Written informed consent was obtained from all patients for photographic and medical record usage. Ideal candidates present with mild-to-moderate jowl ptosis and marionette lines without severe tissue laxity. 8 Patients are positioned supine with slight chin elevation, allowing natural upward migration of ptotic tissues ( Figs. 1 and 2 ).

**Fig. 1:**
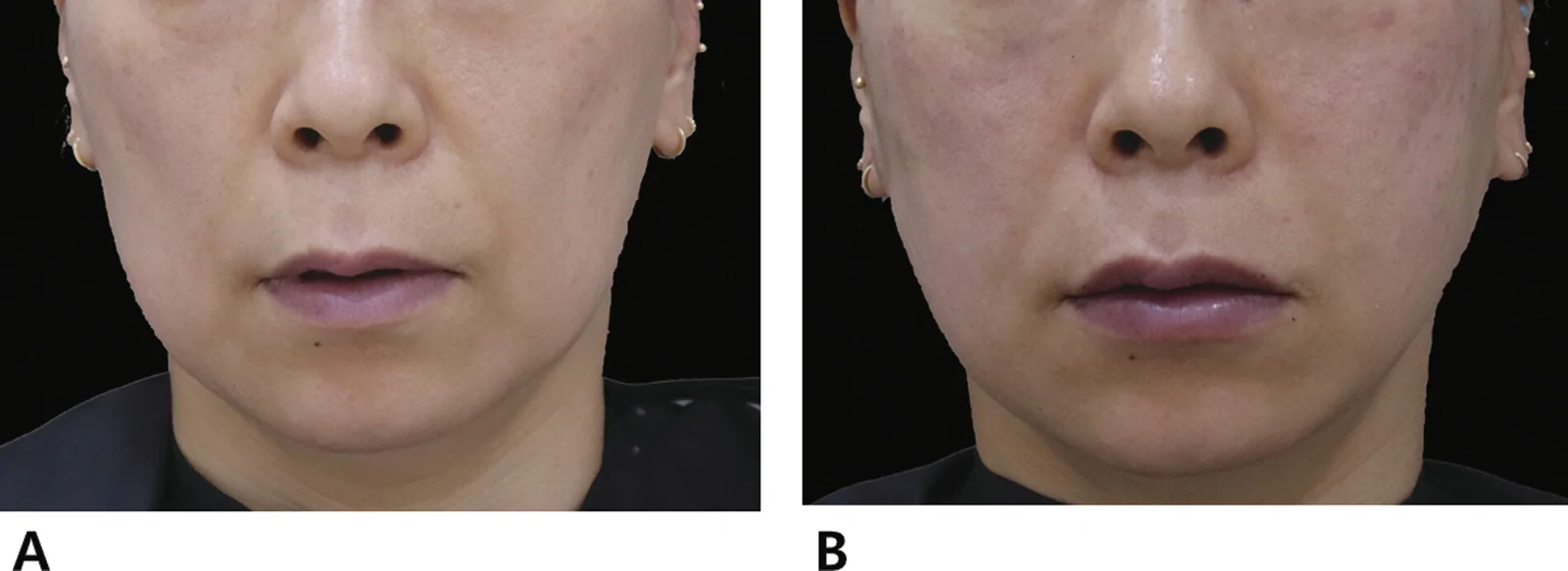
A 40-year-old female with sagging of the jowl fat and a prominent marionette line. Before ( **A** ) and after ( **B** ) treatment using a dual-technique approach with monofilament and bidirectional barbed threads.

**Fig. 2:**
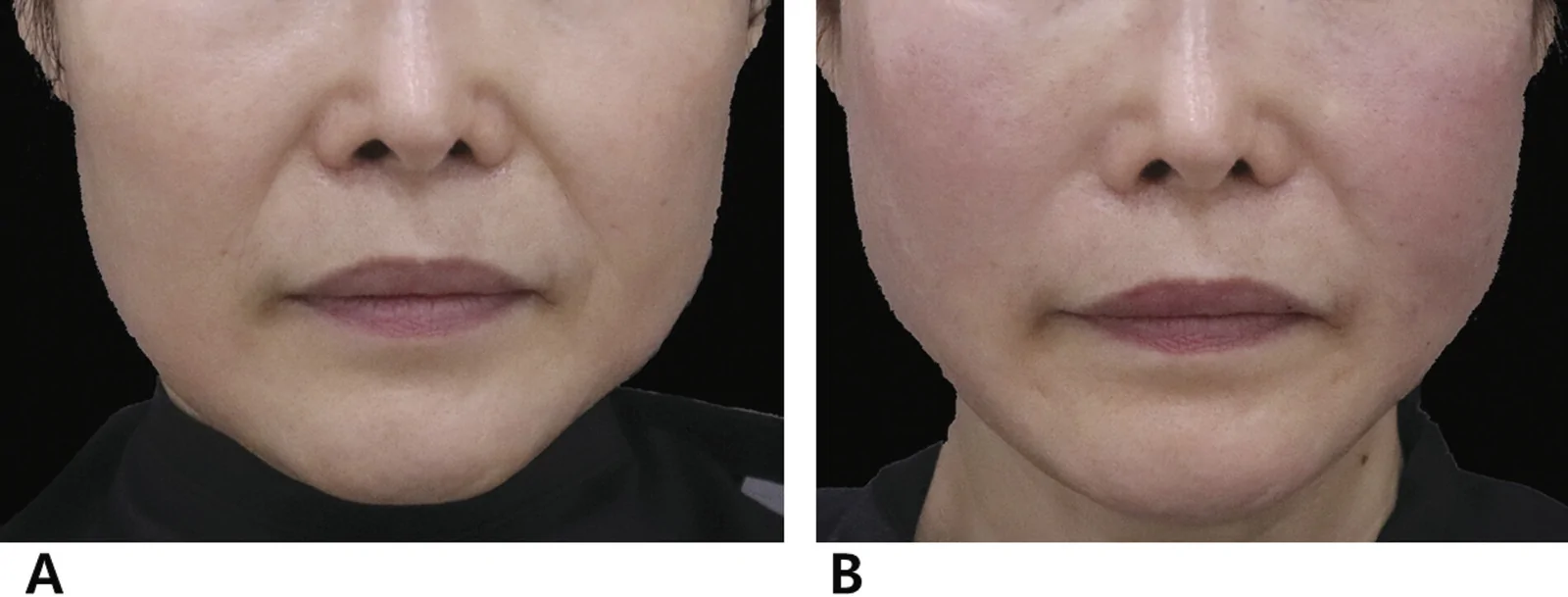
A 47-year-old female with sagging of the jowl fat and a prominent marionette line. Before ( **A** ) and after ( **B** ) treatment using a dual-technique approach with monofilament and bidirectional barbed threads.

### Monofilament Thread Technique

Following structural repositioning, residual fine lines are addressed with volumizing twisted monofilament threads (Sihler Volume, 21G × 60 mm). 6 These are inserted in multiple passes above the marionette line in a cross-hatched pattern ( Fig. 3 and Supplementary Video 1 [available in the online version only]).

**Fig. 3:**
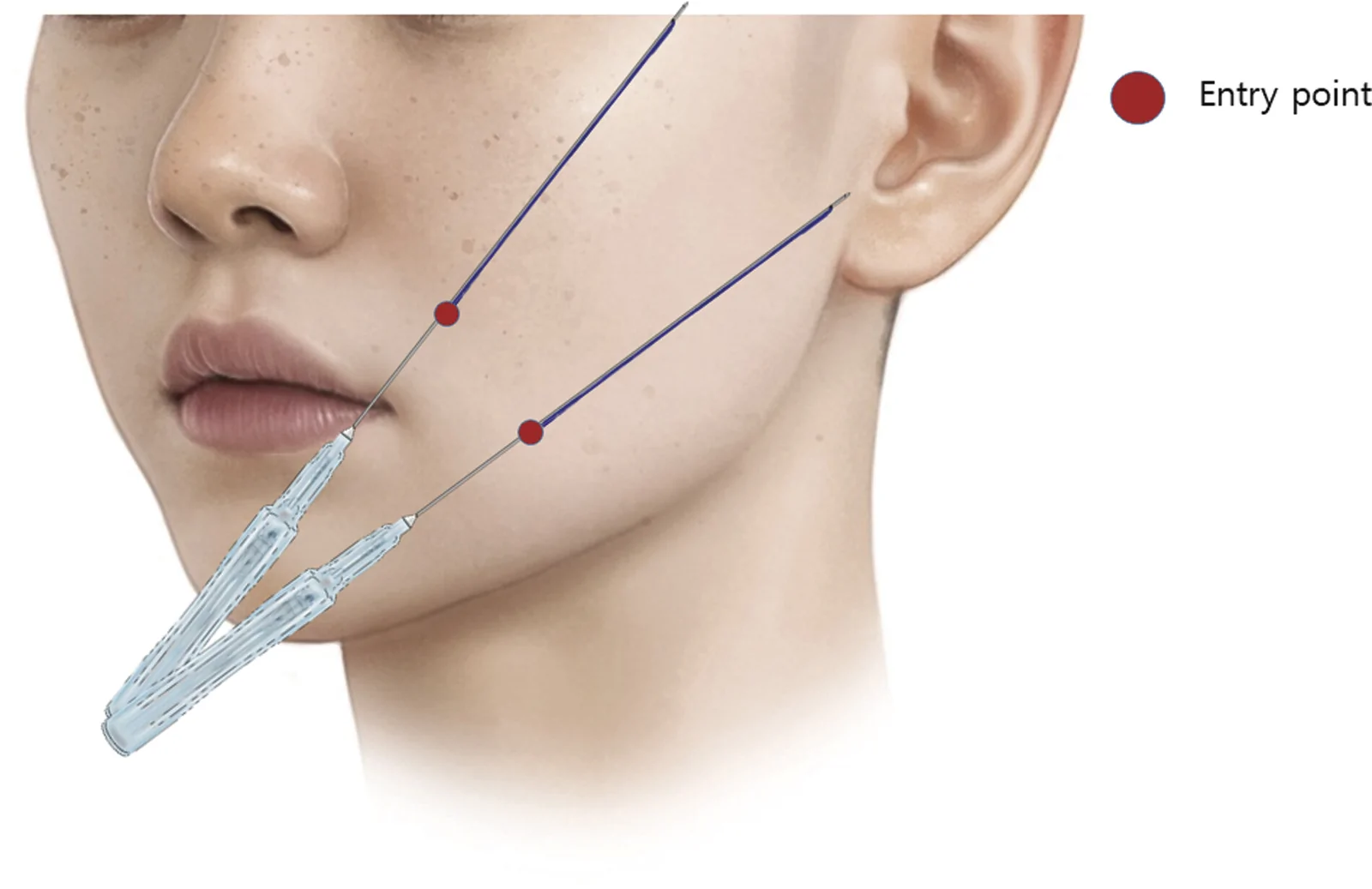
Barbed thread insertion technique. The entry point is positioned above the nasolabial fold, and the thread is directed toward the earlobe for anchoring. The diagram depicts the sub-SMAS plane trajectory engaging the platysma auricular fascia and masseteric-cutaneous ligaments. Demonstration of dual-technique thread-lifting procedure. Barbed thread insertion perpendicular to marionette lines (sub-SMAS plane) followed by monofilament placement parallel to crease lines. (Video recorded and edited by the authors.)

After needle puncture, threads are inserted into subcutaneous tissue superior to the orbicularis oris muscle, advancing inferior-to-superior along the crease line. 9 11 Placement slightly lateral to the crease addresses tissue plane discrepancy from underlying ligamentous attachments. While parallel insertion is possible, perpendicular placement of multiple monofilaments stimulates collagen production along the thread vector, enhancing tissue firmness and reducing skin depression.

### Barbed Thread Technique


**Supplementary Video 1**


The optimal vector for barbed thread insertion is perpendicular to the marionette line, directed toward the auricular region. 6 9 The entry point is positioned above the nasolabial fold, and the thread is directed toward the earlobe for anchoring. Loré's fascia, anterior to the tragus, serves as the anchoring point. 8 10 Loré's fascia was selected as the anchoring point due to its unique advantages for marionette line correction: (1) It provides a perpendicular vector to the marionette line when directed toward the auricular region, (2) it offers consistent fascial thickness for secure engagement, and (3) it avoids the temporal branch of the facial nerve. Compared to temporal fascia anchoring, this approach requires a shorter dissection path; compared to periosteal anchoring, it permits greater tissue mobilization with lower risk of palpable irregularities.

After establishing entry at the anterior tragal margin, the cannula advances until resistance indicates penetration through firm fascial tissue, signifying sub-superficial musculoaponeurotic system (SMAS) plane entry. Within the lateral face, the cannula traverses the thick SMAS layer. As it progresses beyond the lateral orbital rim, transitioning to the anterior facial compartment, SMAS thickness decreases, and tissue laxity increases. 10 The cannula advances with subtle superior angulation to engage ptotic jowl fat, which may be manually elevated during insertion. Unlike nasolabial procedures, jowl elevation requires active skin engagement anterior to the marionette line for adequate superior displacement.

Reversed bidirectional barbed threads (Sihler Threads, 19G or 21G cannula, 100 mm length, 160 mm thread) are inserted through the established entry point. 8 Thread gauge selection depends on skin thickness and ptosis severity—21G threads (Sihler Thread, 21G × 100 mm) may be employed for milder cases requiring less aggressive lifting ( Fig. 4 and Supplementary Video 1 [available in the online version only]).

**Fig. 4:**
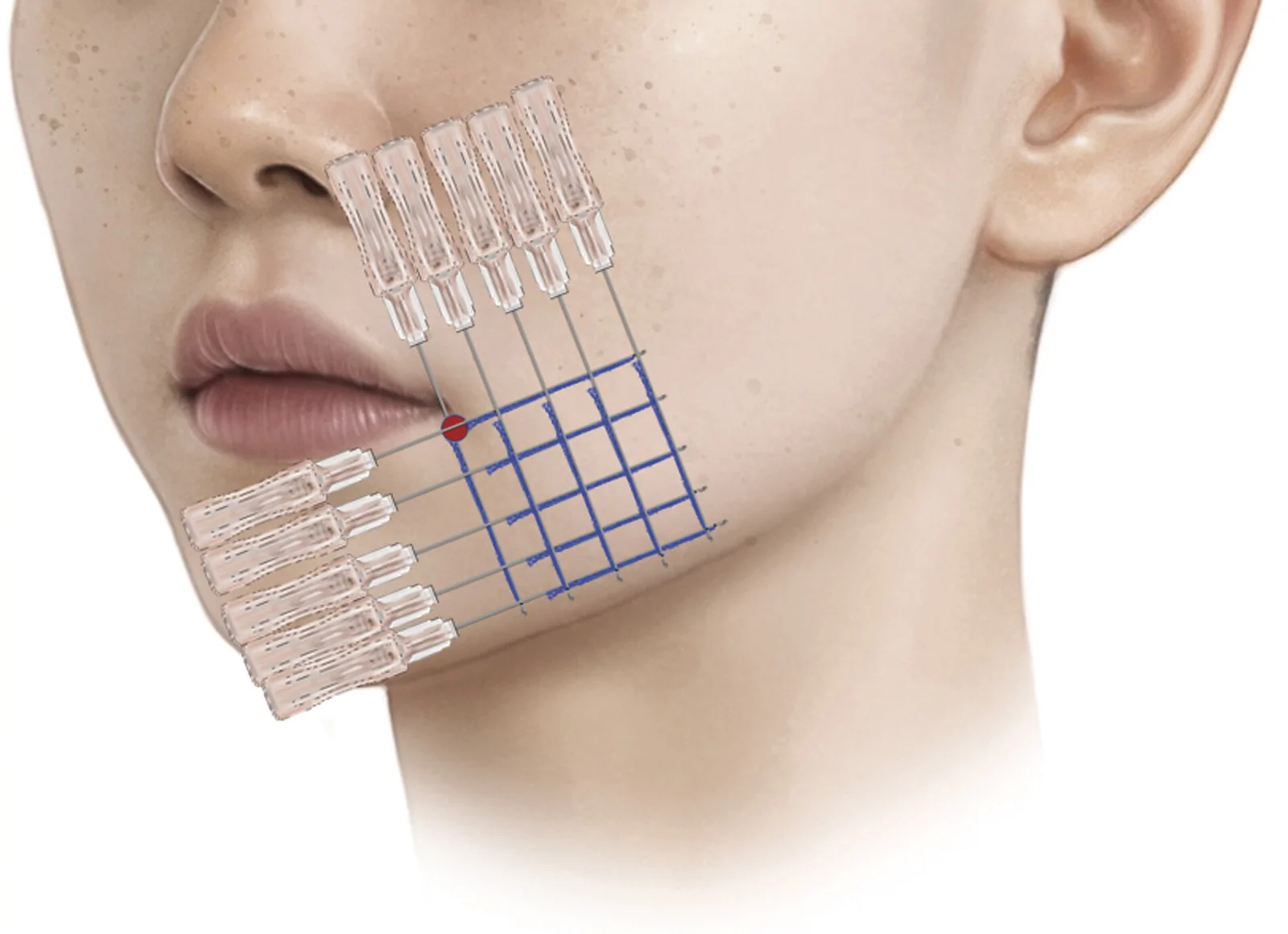
Twisted monofilament thread placement. Threads are positioned in a cross-hatched pattern above the jowl fat.

Resistance at Loré's fascia is differentiated from superficial fascial planes by three tactile cues: (1) a characteristic “pop” sensation as the cannula penetrates the firm fascial layer, (2) increased resistance to advancement requiring gentle pressure, and (3) stability of the cannula position upon release—if the cannula remains securely in position without sagging, correct fascial engagement is confirmed. Depth is standardized by advancing until the cannula hub reaches the skin entry point, ensuring consistent sub-SMAS placement. To prevent asymmetry, bilateral procedures are performed sequentially with continuous comparison; the contralateral side is adjusted to match the first side's thread tension and tissue elevation, with patients assessed in upright position mid-procedure if needed.

The procedural area contains ligamentous structures, including platysma auricular fascia and masseteric-cutaneous ligaments. 10 12 Engaging these structures with the SMAS layer maximizes tissue repositioning effect and procedure longevity.

### Safety Considerations

The perioral region's complex neurovascular anatomy demands exceptional vigilance. Critical structures requiring avoidance include facial artery branches ascending toward the nasolabial fold and traversing superior and inferior to the oral commissure, plus the premasseteric branch originating near the mandibular border and coursing along the masseter's anterior margin. 12 Gentle cannula maneuvering through vascular territories is paramount.

During monofilament insertion, particular caution is essential medial to the marionette line to avoid the mental neurovascular bundle emerging from the mental foramen, typically located midway along the vertical commissure-to-mandible axis. 13

### Adjunctive Treatments

Contemporary perioral rejuvenation benefits from multimodal strategies. 11 14 Botulinum toxin administration to the DAO reduces downward traction on the oral commissure, 14 synergistically complementing barbed thread lifting. This aligns with muscle-balancing paradigms that selectively weaken depressors to optimize resting facial aesthetics. Additional interventions include dermal filler augmentation of pre-marionette depression and prejowl sulcus, volume-reduction procedures for ptotic fat, and additional monofilament threads for fine line attenuation with elasticity enhancement. 11

## Discussion

This dual-technique innovation addresses the unique anatomical considerations of Asian populations through strategic thread placement and anchoring. 1 2 6 9 The inferolateral modiolus position and characteristic fat compartment distribution in Asians necessitate modified treatment protocols compared to Western populations.

### Technical Advantages

Anchoring at Loré's fascia provides stable support for tissue repositioning, while sub-SMAS plane engagement ensures effective mobilization of ptotic tissues. 8 10 The perpendicular insertion vector relative to marionette lines optimally counteracts gravitational descent. Engagement of platysma auricular fascia and masseteric-cutaneous ligaments enhances longevity by leveraging native supporting structures. 12

### Individualized Approach

Combining barbed threads for deep structural repositioning with monofilament threads for superficial enhancement allows comprehensive correction of both structural and surface aging changes. 8 10 11 Thread gauge selection (19G for significant ptosis, 21G for milder cases) tailors intervention intensity to individual tissue characteristics. The infracommissural region permits procedural flexibility while maintaining meticulous technique to avoid neurovascular structures ( Figs. 1 and 2 ).

### Safety Profile

Meticulous adherence to anatomical landmarks and gentle cannula maneuvering through vascular territories is paramount for minimizing adverse events. The described technique specifically addresses the mental neurovascular bundle and facial artery branches—critical structures in the perioral region 13 —and proper identification of these structures prior to thread insertion is essential.

### Limitations and Considerations

Thread-lifting procedures have inherent limitations, including potential thread migration, variable individual collagen response, and the necessity for careful patient selection. Selection criteria should be based on tissue laxity degree and realistic outcome expectations. Severe tissue laxity may require alternative or combined surgical approaches. Long-term outcomes depend on proper technique execution and adjunctive treatment integration.

### Clinical Application

This approach is particularly suited for Asian patients presenting with mild-to-moderate marionette lines and jowl ptosis who desire minimally invasive intervention. The technique can be performed under local anesthesia with minimal downtime. Integration with botulinum toxin and dermal fillers provides comprehensive perioral rejuvenation addressing both dynamic and static components.

### Conclusion

This dual-technique thread-lifting approach offers an effective, minimally invasive solution for marionette lines and jowl ptosis in Asian populations. 6 8 9 By addressing unique anatomical variations—particularly the inferolateral modiolus position—and combining structural barbed threads with superficial monofilament threads, the technique achieves comprehensive correction while preserving natural facial dynamics. Proper anatomical understanding, precise technique execution, and integration with adjunctive treatments optimize outcomes. 11 14 Future comparative studies with larger patient cohorts and longer follow-up periods would further validate this approach and refine patient selection criteria.
